# Supplementary material for: Risk Assessment Model System for Aquatic Animal Introduction Based on Analytic Hierarchy Process (AHP)
Source: Animals (Basel). 2023 Jun 19;13(12):2035. doi: 10.3390/ani13122035 (PMC10294931; doi:10.3390/ani13122035)
Supplement: Supplementary file 1 [file animals-13-02035-s001.zip › animals-2413858-supplementary.pdf]

Supplementary Material

Table S1. Discussion of tertiary indexes of *Pterygoplichthys pardalis*.

Indexes	Content of the Discussion
p11	*Pterygoplichthys pardalis* has been explicitly listed as an invasive species by the Chinese government. Therefore, the risk of biological invasion is extremely high.
p12	*Pterygoplichthys pardalis* is not listed on the IUCN Red List of Threatened Species; it is not an endangered species.
p23	*Pterygoplichthys pardalis* are omnivorous fish with a large appetite. Algae, benthos, fish eggs, etc., can become food for *Pterygoplichthys pardalis*. *Pterygoplichthys pardalis* can also destroy the habitats of small aquatic animals when foraging, which will pose a threat to native species.
p31	The exporting country of *Pterygoplichthys pardalis* is Brazil, and the trade situation between Brazil and China in the field of animal and plant agricultural products has been stable for a long time.
p32	The national fisheries and medical management agencies of both countries have sufficient experience and ability to be responsible for diseases related to the *Pterygoplichthys pardalis.*
p33	The relevant fisheries and medical management organizations in the main cultivation areas of *Pterygoplichthys pardalis* in Brazil have sufficient experience and ability to prevent and control diseases related to *Pterygoplichthys pardalis*.
p34	Introduction is supported by relevant materials, such as *the List of Quarantine Diseases for the Animals Imported to the People's Republic of China*, The Office International des Epizooties (OIE)'s *Diagnostic Manual of Aquatic Animals Diseases*, and national policy documents.
p35	Stakeholders need to fulfill their obligations to prevent and control diseases related to *Pterygoplichthys pardalis*.
p36	The relevant fisheries and medical diagnostic laboratories in both countries have the ability to diagnose, detect, treat, and prevent diseases related to *Pterygoplichthys pardalis*.
p37	The medical diagnostic laboratories at all levels in Brazil have the ability to diagnose, detect, treat, and prevent diseases related to *Pterygoplichthys pardalis*.
p38	The reference laboratories are highly recognized by both countries and managed in an orderly manner.
p39	The governments of both countries would conduct an investigation and provide financial support for the specific situation of related diseases of *Pterygoplichthys pardalis*.
p43	In the past five years, there has been no large-scale disease outbreak in *Pterygoplichthys pardalis* cultivation farms in Brazil.
p51	The Office International des Epizooties (OIE)'s *Diagnostic Manual of Aquatic Animals Diseases* will be updated with endemic diseases and susceptible diseases as soon as possible.
p52	If there is an outbreak, measures will be taken promptly, relevant areas will be immediately reported and sealed off, and doctors specializing in fish will conduct testing and diagnosis.
p53	Both countries have very effective management measures in specific epidemic areas and biosafety isolation areas with orderly management.
p54	Both countries have strict animal epidemic surveillance plans and strict implementation plans.
p55	Both countries have strict vector animal monitoring plans and strict implementation.
p56	Regular feeding, partly with drug formula feed, can improve the immunity of *Pterygoplichthys pardalis* to some diseases.
p57	All biological data related to the introduction of *Pterygoplichthys pardalis* germplasm resources can be traced throughout the process.
p58	The relevant departments at all levels of both countries shall ensure that the *Pterygoplichthys pardalis* cultured in the cultivation farms meets the biosafety requirements.
p59	The relevant ports of both countries have strict quarantine measures to prevent the invasion of diseases.
p62	*Pterygoplichthys pardalis* is sold mainly as ornamental fish in China. Although the aquarium trade is accompanied by huge profits, *Pterygoplichthys pardalis* has been included in the list of China's national key regulatory species. If it is not properly regulated, it will cause serious biological invasion and

	socioeconomic losses.
p71	Common pathogens that can infect *Pterygoplichthys pardalis* include *Ichthyophthirius multifiliis*, *Flavobacterium columnaris*, *A. punotata* f.*intestinalis*, and *Saprolegnia*, *Achlya*, all of which are highly pathogenic.
p74	The vast majority of *Pterygoplichthys pardalis* diseases are nonpathogenic to humans.
p91	The infection, incidence, and mortality rates of the above pathogens are extremely high, and most of the diseased fish will have increased skin mucus and a layer of dense white spots on the body, gill ulceration and difficulty breathing, and other symptoms, such as abnormal swimming. Even if there is no death, they will lose their commercial value.
p92	The main infection sites include the body surface, gills, liver, spleen, kidney, and fish eggs.
p93	According to histopathological observations, most pathogens induce severe necrosis of organs or tissues in fish, with necrotic foci appearing.
p94	The host of most pathogens, *Pterygoplichthys pardalis*, from the juvenile to adult stages, can become infected.
p101	Currently, there is no effective vaccine available for large-scale use against the susceptible disease of *Pterygoplichthys pardalis*.
p102	Currently, there are effective diagnostic and detection methods for the aforementioned pathogens.
p103	For most pathogens, the existing epidemic prevention and control measures can control the disease risk within a certain range.
p104	For most pathogens, there is yet no feasible treatment method; some diseases can be prevented and controlled only at a preventive level.

Table S2. Discussion of tertiary indexes of *Macrobrachium rosenbergii*.

Indexes	Content of the Discussion
p11	*Macrobrachium rosenbergii* is an economic shrimp, not an invasive alien species. However, large-scale cultivation of *Macrobrachium rosenbergii* also has a certain risk of biological invasion.
p12	*Macrobrachium rosenbergii* is not listed on the IUCN Red List of Threatened Species, so it is not an endangered species.
p23	*Macrobrachium rosenbergii* is an omnivorous shrimp that feeds mainly on small crustaceans, insects, and some organic debris and has a large food intake.
p31	The exporting country of *Macrobrachium rosenbergii* is Thailand. As an important country in Southeast Asia, Thailand is also a friendly neighbor of China. In the context of economic globalization, Thailand has become one of the trading countries with close relations with China.
p32	The national fisheries and medical management agencies of both countries have sufficient experience and ability to take charge of diseases related to *Macrobrachium rosenbergii*.
p33	The relevant fisheries and medical management organizations in the main cultivation areas of *Macrobrachium rosenbergii* in Thailand have sufficient experience and ability in preventing and controlling diseases related to *Macrobrachium rosenbergii*.
p34	Introduction is supported by relevant materials, such as *the List of Quarantine Diseases for the Animals Imported to the People's Republic of China*, The Office International des Epizooties (OIE)'s *Diagnostic Manual of Aquatic Animals Diseases*, and national policy documents.
p35	Stakeholders need to fulfill their obligations to prevent and control diseases related to *Macrobrachium rosenbergii*.
p36	The relevant fisheries and medical diagnostic laboratories have the ability to diagnose, detect, treat, and prevent diseases related to *Macrobrachium rosenbergii*.
p37	The medical diagnostic laboratories at all levels in Thailand have the ability to diagnose, detect, treat, and prevent diseases related to *Macrobrachium rosenbergii*.
p38	The reference laboratories are highly recognized by both countries and managed in an orderly manner.
p39	The governments of both countries would conduct an investigation and provide financial support for the specific situation of related diseases of *Macrobrachium rosenbergii*.
p43	In the past five years, there has been no large-scale disease outbreak in *Macrobrachium rosenbergii* cultivation farms in Thailand.
p51	The Office International des Epizooties (OIE)'s *Diagnostic Manual of Aquatic Animals Diseases* will be updated with endemic diseases and susceptible diseases as soon as possible.
p52	If there is an outbreak, measures will be taken promptly, relevant areas will be immediately reported and sealed off, and doctors specializing in fish will conduct testing and diagnosis.
p53	Both countries have very effective management measures in specific epidemic areas and biosafety isolation areas with orderly management.
p54	Both countries have strict animal epidemic surveillance plans and strict implementation plans.
p55	Both countries have strict vector animal monitoring plans and strict implementation.
p56	Regular feeding with part of the drug formula feed can improve the immune ability of *Macrobrachium rosenbergii* against some diseases.
p57	All biological data related to the introduction of *Macrobrachium rosenbergii* germplasm resources can be traced throughout the process.
p58	The relevant departments at all levels of both countries shall check and ensure that the *Macrobrachium rosenbergii* cultured in the cultivation farms meet the biosafety requirements.
p59	The relevant ports of both countries have strict quarantine measures to prevent the invasion of diseases.
p62	*Macrobrachium rosenbergii* is one of the more widely cultured freshwater shrimps in southern China and has generated enormous economic benefits. However, while generating enormous economic benefits, its economic losses caused by diseases can also cause great harm to the aquaculture industry in China.
p71	The main diseases of *Macrobrachium rosenbergii* include decapod iridovirus disease, cloudy muscle disease, and larval syndrome. The pathogens include bacteria, viruses, and other types, which can cause great harm.

p74	The vast majority of *Macrobrachium rosenbergii* diseases are nonpathogenic to humans.
p91	The infection rate and the incidence and mortality rates of the above diseases are very high; they are accompanied by reduced feeding, slow reaction, limb redness, and other phenomena. Even if there is no death, its commercial value has been completely lost.
p92	The main infection sites include the body surface, gills, liver, pancreas, and other parts.
p93	According to histopathological observations, most pathogens directly invade the organs or tissues of *Macrobrachium rosenbergii*, causing severe necrosis and necrotic foci.
p94	*Macrobrachium rosenbergii* can be infected from larva to adult, but the morbidity and mortality of the larva are higher than those of the adult.
p101	Currently, there is no inexpensive and effective vaccine for the above diseases that can be used on a large scale.
p102	Most pathogens can be detected via molecular biological methods, microscopic electron microscopy, and other methods.
p103	For most pathogens, there are practical and effective prevention and control measures.
p104	For most pathogens, there is no effective treatment, and control can only be carried out at the preventive level.

Table S3. Discussion of tertiary indexes of *Crassostrea gigas*.

Indexes	Content of the Discussion
p11	*Crassostrea gigas* is an economic shellfish, not an invasive alien species. However, large-scale cultivation of *Crassostrea gigas* also has a certain risk of biological invasion.
p12	*Crassostrea gigas* is not listed on the IUCN Red List of Threatened Species, so it is not an endangered species.
p23	*Crassostrea gigas* feed mainly on diatoms and other organic debris, but their intake is large, which may change the species composition and quantity of zooplankton or phytoplankton in the water thereby affecting the environment.
p31	The exporting country of *Crassostrea gigas* is Japan; as a developed country in East Asia, Japan's agricultural trade with China is relatively stable state.
p32	The national fisheries and medical management agencies of both countries have sufficient experience and ability to take charge of diseases related to *Crassostrea gigas*.
p33	The relevant fisheries and medical management organizations in the main cultivation areas of *Crassostrea gigas* in Japan have sufficient experience in preventing and controlling diseases related to *Crassostrea gigas*.
p34	Introduction is supported by relevant materials, such as *the List of Quarantine Diseases for the Animals Imported to the People's Republic of China*, The Office International des Epizooties (OIE)'s *Diagnostic Manual of Aquatic Animals Diseases*, and national policy documents.
P35	Stakeholders need to fulfill their obligations to prevent and control diseases related to *Crassostrea gigas*.
p36	The relevant fisheries and medical diagnostic laboratories have the ability to diagnose, detect, treat, and prevent diseases related to *Crassostrea gigas*.
p37	The medical diagnostic laboratories at all levels in Japan have the ability to diagnose, detect, treat, and prevent diseases related to *Crassostrea gigas*.
p38	The recognition of reference laboratories by both countries can ensure the smooth progress of relevant work.
p39	The governments of both countries would investigate the specific situation of diseases related to *Crassostrea gigas* and provide financial support.
p43	*Crassostrea gigas* cultivation farms in neither country have experienced large-scale outbreaks of disease in the past five years.
p51	The Office International des Epizooties (OIE)'s *Diagnostic Manual of Aquatic Animals Diseases* will be updated with endemic diseases and susceptible diseases as soon as possible.
p52	If there is an outbreak, measures will be taken promptly, relevant areas will be immediately reported and sealed off, and doctors who specialize in fish will conduct testing and diagnosis.
p53	Both countries have very effective management measures in specific epidemic areas and biosafety isolation areas with orderly management.
p54	Both countries have strict animal epidemic surveillance plans and strict implementation plans.
p55	Both countries have strict vector animal monitoring plans and strict implementation.
p56	Pacific oysters have strong adaptability, but there is currently no method by which to significantly improve the immunity of *Crassostrea gigas*.
p57	All biological data related to the introduction of *Crassostrea gigas* germplasm resources can be traced throughout the process.
p58	The relevant departments at all levels of both countries shall check and ensure that the *Crassostrea gigas* cultured in the cultivation farms meet the biosafety requirements.
p59	The relevant ports of both countries have strict quarantine measures to prevent the invasion of diseases.
p62	*Crassostrea gigas*, as one of the main economic shellfish in the South China Sea, has generated enormous economic benefits. However, the economic losses caused by the outbreak of related diseases will also cause great harm to the shellfish aquaculture industry in China.
p74	Common pathogens that can infect *Crassostrea gigas* include oyster velar virus (OVV), herpes-type virus, *Perkins marinus*, and *Vibrio* sp., all of which are highly pathogenic.
p91	Most *Crassostrea gigas* diseases are nonpathogenic to humans.

p92	*Crassostrea gigas* infected with OVV have decreased activity, with visceral masses contracting into the shell and epithelial cells on the shell lose flagella, and some cells become separated and shed; the digestive glands of *Crassostrea gigas* infected with herpes-type virus turn pale gray; *Perkins marinus* can parasitize and destroy all soft tissues throughout the *Crassostrea gigas* body. If *Crassostrea gigas* are infected with these pathogens, they will lose their commercial value.
p93	The pathogens mentioned above mainly harm the connective tissue, adductor muscle, epithelial tissue of the digestive system, viscera, and blood vessels of *Crassostrea gigas*.
p94	Through histopathological observation, it was found that most pathogens penetrated into various organs in the *Crassostrea gigas* body, ultimately leading to *Crassostrea gigas* death.
p101	Currently, there is no widely used vaccine against diseases related to *Crassostrea gigas*.
p102	Currently, there are very effective and reliable detection and diagnosis methods for *Crassostrea gigas* diseases.
p103	Currently, there are many practical and effective prevention measures for *Crassostrea gigas* diseases.
p104	For most pathogens, there is no effective treatment, and control can be carried out only at the preventive level.

Table S4. **Discussion of tertiary indexes** of *Trachemys scripta elegans*.

Indexes	Content of the Discussion
p11	*Trachemys scripta elegans* has been listed by IUCN as one of the 100 most destructive invasive species. Therefore, the risk of biological invasion is high.
p12	*Trachemys scripta elegans* is not listed on the IUCN Red List of Threatened Species, so it is not an endangered species.
p23	The *Trachemys scripta elegans* is an omnivorous tortoise that likes to eat small fish and vegetable leaves. The *Trachemys scripta elegans* can plunder the survival resources of other organisms.
p31	The exporting country of *Trachemys scripta elegans* is the United States. As a large agricultural country, the United States has maintained a relatively stable agricultural trade with China.
p32	The national fisheries and medical management agencies of both countries have sufficient experience and ability to be responsible for diseases related to the *Trachemys scripta elegans*.
p33	The relevant fishery and medical management organizations in the main cultivation areas of *Trachemys scripta elegans* in the US have sufficient experience in preventing and controlling diseases related to *Trachemys scripta elegans*.
p34	Introduction is supported by relevant materials, such as *the List of Quarantine Diseases for the Animals Imported to the People's Republic of China*, The Office International des Epizooties (OIE)'s *Diagnostic Manual of Aquatic Animals Diseases*, and national policy documents.
p35	Stakeholders need to fulfill their obligations to prevent and control diseases related to *Trachemys scripta elegans*.
p36	The relevant fisheries and medical diagnostic laboratories have the ability to diagnose, detect, treat, and prevent diseases related to *Trachemys scripta elegans*.
p37	The medical diagnostic laboratories at all levels in the US have the ability to diagnose, detect, treat, and prevent diseases related to *Trachemys scripta elegans*.
p38	The recognition of reference laboratories by both countries can ensure the smooth progress of relevant work.
p39	The governments of both countries would conduct an investigation and provide financial support for the specific situation of related diseases of *Trachemys scripta elegans*.
p43	In the past five years, there has been no large-scale disease outbreak in *Trachemys scripta elegans* cultivation farms in the United States.
p51	The Office International des Epizooties (OIE)'s *Diagnostic Manual of Aquatic Animals Diseases* will be updated with endemic diseases and susceptible diseases as soon as possible.
p52	If there is an outbreak, measures will be taken promptly, relevant areas will be immediately reported and sealed off, and doctors who specialize in fish will conduct testing and diagnoses.
p53	Both countries have very effective management measures in specific epidemic areas and biosafety isolation areas with orderly management.
p54	Both countries have strict animal epidemic surveillance plans and strict implementation plans.
p55	Both countries have strict vector animal monitoring plans and strict implementation.
p56	The *Trachemys scripta elegans* has a strong adaptive ability, but there are currently no reports related to improving the immunity of the *Trachemys scripta elegans*.
p57	All biological data related to the introduction of *Trachemys scripta elegans* germplasm resources can be traced throughout the process.
p58	The relevant departments at all levels of both countries shall check and ensure that the *Trachemys scripta elegans* cultured in the cultivation farms meet the biosafety requirements.
p59	The relevant ports of both countries have strict quarantine measures to prevent the invasion of diseases.
p62	*Trachemys scripta elegans* is one of the main cultured tortoises in China. However, because of its wide-ranging food habits, its strong adaptability, and the transmission medium of some pathogens, relevant departments must pay attention to the introduction and culturing supervision of *Trachemys scripta elegans*. If *Trachemys scripta elegans* causes biological invasion or disease outbreak accidentally, it can cause great harm to the ecosystem and aquaculture industry.

p71	Common diseases of *Trachemys scripta elegans* include neck ulcer of the tortoise, shell ulcer of the tortoise, and ulcerous shell disease of the tortoise. Most diseases are caused by the combined action of various bacteria, fungi, viruses, and other factors, as well as the deterioration of water quality during aquaculture.
p74	Most pathogens of tortoise disease pose no threat to human health.
p91	The infection rate and incidence rate of the above diseases are extremely high. The tortoises suffering from neck ulcer disease will have a swollen neck base, gray ring spots, neck ulceration, and other symptoms; tortoises suffering from shell ulcer disease will have necrosis, whitening, or erosion of the skin tissue in the ventral shell, limbs, and tail; the shell of tortoises suffering from ulcerous shell disease undergoes erosion, with severe perforation and visible muscles. Most of the diseased tortoises have reduced activity and eventually die.
p92	The above diseases affect mainly the neck, limbs, shell, and other parts of the tortoise.
p93	The infected site of the disease usually undergoes ulceration and inflammation. According to histopathological observations, pathogenic bacteria usually invade various tissues of the diseased tortoise.
p94	Both young and adult tortoises can become sick, but the infection and prevalence rates of young tortoises are higher than those of adult tortoises.
p101	Currently, there is no widely used vaccine for diseases related to *Trachemys scripta elegans*.
p102	Currently, there are very effective and reliable detection and diagnosis methods for diseases related to *Trachemys scripta elegans*.
p103	Currently, there are many practical and effective prevention measures for the disease of *Trachemys scripta elegans*.
p104	For most pathogens, there is no effective treatment, and control can be carried out only at the preventive level.

Table S5. **Discussion of tertiary indexes** of *Ambystoma mexicanum*.

Indexes	Content of the Discussion
p11	*Ambystoma mexicanum* are sold mainly as pets, and they are not an invasive alien species.
p12	Currently, *Ambystoma mexicanum* has been included in the IUCN Red List of Threatened Species (critically endangered, CR), but artificial cultivation has been achieved in China, so the risk of *Ambystoma mexicanum* is relatively low.
p23	*Ambystoma mexicanum* is an omnivorous amphibian that feeds mainly on algae, earthworms, insects, small fish, and shrimp.
p31	The exporting country of *Ambystoma mexicanum* is Mexico, and the agricultural product trade between Mexico and China has been stable for a long time.
p32	The national fisheries and medical management agencies of both countries have sufficient ability and will be responsible for diseases related to *Ambystoma mexicanum*.
p33	The relevant fisheries and medical management agencies of both countries have sufficient experience in the prevention and control of diseases of the *Ambystoma mexicanum*.
p34	Introduction is supported by relevant materials, such as *the List of Quarantine Diseases for the Animals Imported to the People's Republic of China*, The Office International des Epizooties (OIE)'s *Diagnostic Manual of Aquatic Animals Diseases*, and national policy documents.
p35	Stakeholders need to fulfill their obligations to prevent and control diseases related to the *Ambystoma mexicanum*.
p36	The relevant fisheries and medical diagnostic laboratories have the ability to diagnose, detect, treat, and prevent diseases related to *Ambystoma mexicanum*.
p37	The medical diagnostic laboratories at all levels in Mexico have the ability to diagnose, detect, treat, and prevent diseases related to *Ambystoma mexicanum*.
p38	The recognition of reference laboratories by both countries can ensure the smooth progress of relevant work.
p39	The governments of both countries would conduct an investigation and provide financial support for the specific situation of related diseases of *Ambystoma mexicanum*.
p43	In the past five years, there has been no large-scale disease outbreak in *Ambystoma mexicanum* cultivation farms in the Mexico.
p51	The Office International des Epizooties (OIE)'s *Diagnostic Manual of Aquatic Animals Diseases* will be updated with endemic diseases and susceptible diseases as soon as possible.
p52	If there is an outbreak, measures will be taken promptly, relevant areas will be immediately reported and sealed off, and doctors who specialize in fish will conduct testing and diagnosis.
p53	Both countries have very effective management measures in specific epidemic areas and biosafety isolation areas with orderly management.
p54	Both countries have strict animal epidemic surveillance plans and strict implementation plans.
p55	Both countries have strict vector animal monitoring plans and strict implementation.
p56	The main pathogens that infect *Ambystoma mexicanum* are bacteria, fungi, and parasitic animals. Currently, there are few reports on improving the immunity of the *Ambystoma mexicanum*.
p57	All biological data related to the introduction of *Ambystoma mexicanum* germplasm resources can be traced throughout the process.
p58	The relevant departments at all levels of both countries ensure that the *Ambystoma mexicanum* cultured in the cultivation farms meet the biosafety requirements.
p59	The relevant ports of both countries have strict quarantine measures to prevent the invasion of diseases.
p62	*Ambystoma mexicanum* is distributed mainly in southern Mexico. Because of the overexploitation of local resources, the *Ambystoma mexicanum* has become an endangered species. However, in China, *Ambystoma mexicanum* is able to be bred artificially, but the cost of artificial breeding is high, and as a wild resource, *Ambystoma mexicanum* is still precious. Therefore, the introduction and cultivation of *Ambystoma mexicanum* are also accompanied by significant economic benefits and risks.
p71	The main diseases of *Ambystoma mexicanum* are caused by bacterial or fungal infections or parasitic parasites, presenting symptoms such as water mold, sepsis, and ascites. Most diseases are caused by the

	combined action of multiple bacteria and fungi, and some are caused by water quality deterioration during aquaculture.
p74	Most pathogens pose no threat to human health.
p91	Sick *Ambystoma mexicanum* can experience symptoms such as skin ulceration, red blood spots on the tail, thickened neck, and enlarged body, as well as other symptoms, such as emaciation, slow and weak movement, and anorexia, which can lead to loss of commercial value.
p92	Most diseases mainly affect the skin, tail, and other parts.
p93	The infected site of the disease usually undergoes ulceration and inflammation. According to histopathological observations, the pathogenic bacteria invade various tissues in the body of *Ambystoma mexicanum*, causing necrosis and a large number of necrotic foci.
p94	*Ambystoma mexicanum* can be infected from the larva stage to adulthood, but the mortality rate of the larva is greater than that of the adult.
p101	Currently, there is no widely used vaccine for diseases related to *Ambystoma mexicanum*.
p102	Currently, there are very effective and reliable detection and diagnosis methods for related diseases of *Ambystoma mexicanum*.
p103	Currently, there are many practical and effective prevention measures for the disease of *Ambystoma mexicanum*.
p104	For most pathogens, there is no effective treatment, and control can be carried out only at the preventive level.
